# The Impact of BRAF Mutation on the Recurrence of Papillary Thyroid Carcinoma: A Meta-Analysis

**DOI:** 10.3390/cancers12082056

**Published:** 2020-07-25

**Authors:** Xin Li, Hyungju Kwon

**Affiliations:** Department of Surgery, Ewha Womans University Medical Center, 1071 Anyangcheon-ro, Yangcheon-Gu, Seoul 07985, Korea; yikicat@naver.com

**Keywords:** papillary thyroid carcinoma, recurrence, BRAF mutation

## Abstract

Previous meta-analyses indicated that the BRAF V600E mutation was associated with an increased recurrence rate of papillary thyroid carcinoma (PTC). However, with recent publications of large cohort studies, the need for an updated meta-analysis increases. Therefore, we conducted a comprehensive meta-analysis to assess the impact of the BRAF V600E mutation on PTC recurrences. We performed a literature search using PubMed, SCOPUS, the Cochrane Database of Systematic Reviews, and the Web of Science Core Collection, from their inception to May 31, 2020. The relevant studies compared recurrence rates using the hazard ratio (HR) of BRAF mutations; 11 studies comprising 4674 patients were identified and included. Recurrence rates in patients with the BRAF V600E mutation were comparable with BRAF wild-type patients (HR 1.16, 95% CI 0.78–1.71), after adjustment for possible confounders. In subgroup analysis, both geographical region (HRs for America, Asia, and Europe were 2.16, 1.31 and 0.66, respectively) and tumor stage (HRs for stage I and II were 1.51 and 4.45, respectively) can affect the HRs of the BRAF mutation for recurrence. In conclusion, the BRAF mutation does not increase the risk of recurrences in patients with PTC. Differences in the geographical region or tumor stage should be considered when interpreting the impact of a BRAF mutation on recurrence.

## 1. Introduction

Thyroid cancer is one of the most common cancers in Korea and worldwide, and its incidence has rapidly increased over the past two decades [1]. The incidence of thyroid cancer in Korea increased about 5.8 times from 6.3 cases per 100,000 in 1999 to 36.8 cases per 100,000 in 2017. Papillary thyroid carcinoma (PTC) comprises the vast majority of all thyroid cancers, which is generally associated with favorable outcomes [2,3]. However, some patients present with aggressive disease, including regional or distant metastasis, and experience significant progression [4,5,6]. Recurrence and cancer-related death can occur more than 20 years after the initial diagnosis of PTC [7]. This wide spectrum of clinical behaviors sometimes causes dilemmas in decision making and clinical risk stratification during the management of PTC [8].

Many studies have attempted to differentiate high-risk patients from the population with an excellent prognosis [9,10]. Conventional clinicopathological factors, including age, sex, tumor size, vascular invasion, and lymph node metastasis, have been extensively investigated to predict disease recurrence and mortality. The American Thyroid Association (ATA) first established a three-tiered risk stratification system in 2006 using conventional risk factors [11]. In recent years, as the BRAF V600E mutation has emerged as a powerful predictive factor for PTC recurrence, molecular marker-based risk stratification has been proposed [12]. The latest 2015 ATA system has incorporated molecular markers, including the BRAF V600E mutation [13]. These ATA guidelines indicated that the mutational status of BRAF could assist in proper risk stratification, although they did not recommend routine analysis of BRAF V600E mutations.

Some researchers have conducted meta-analyses to explore the association between the BRAF V600E mutation and recurrence rates of PTC [14,15,16,17,18]. However, all meta-analyses except one used the odds ratio (OR), which only measured the number of recurrences without consideration for when they occur. ORs may be appropriate for measuring dichotomous outcomes, but they are less suitable for analyzing time-to-event outcomes [19]. Time-to-event outcomes, including recurrences, can be more accurately analyzed using hazard ratios (HRs). Only Wang et al. used HRs to investigate the role of the BRAF V600E mutation in the recurrence rates of PTC, but this study had some limitations, such as the inclusion of duplicate studies and inaccurate estimation of HRs in some enrolled studies [20]. Furthermore, several studies using large cohorts have been reported recently after the publication of that meta-analysis [21,22,23,24,25,26,27,28].

Therefore, we performed a systematic review and meta-analysis to assess the impact of the BRAF V600E mutation on PTC recurrences using HRs.

## 2. Results

### 2.1. Search Results

**Figure 1:**
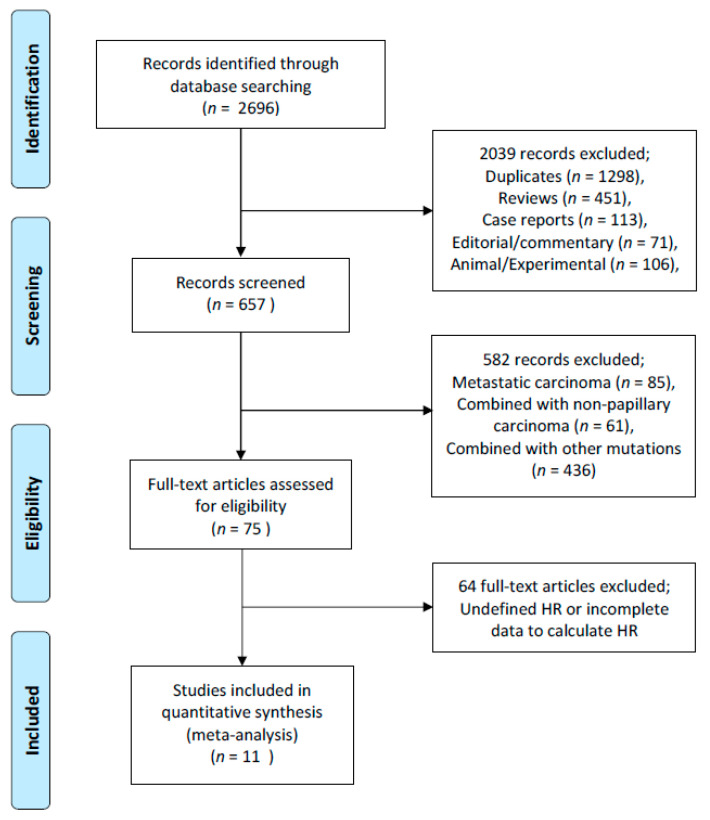
The preferred reporting items for systematic reviews and meta-analyses (PRISMA) flow diagram of the including studies.

The literature search identified 2696 potentially relevant articles, of which 657 were screened for further review (Figure 1). The exclusion reasons were duplicates, non-English articles, reviews, case reports or commentaries and experimental studies. Then, 75 articles were reviewed in full text. By excluding articles with insufficient data to calculate HR, at last, 11 articles were selected for inclusion in this meta-analysis, and their main features are summarized in Table 1.

### 2.2. Effect of the BRAF V600E Mutation in Tumor Recurrence

**Figure 2:**
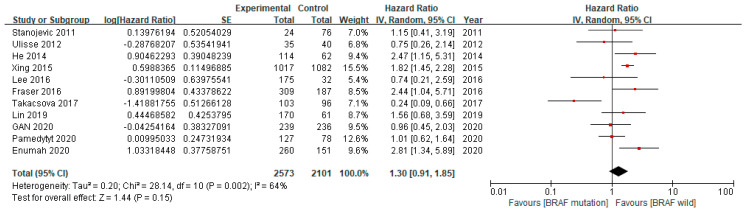
Forest plot of the included studies analyzing the recurrence rates between the patients with the BRAF V600E mutation and those with wild-type BRAF.

Figure 2 shows the meta-analysis findings. Among 4674 patients in 11 studies, 2573 patients with the BRAF V600E mutation were identified, and the median mutation rate was 62.0% (range from 24.0% to 84.5%). The recurrence rate of PTC in the entire group ranged from 5.9% to 34.1%, with an average rate of 18.3%.

The median recurrence rate in the BRAF V600E mutation group was 19.0% (range from 7.5% to 39.5%), while that in the wild-type BRAF group was 10.8% (range from 4.2% to 33.3%). We used random-effects models, as moderate heterogeneity was observed across the studies (I^2^ = 62%, *p* = 0.003). Compared to the wild-type BRAF patients, the patients with the BRAF V600E mutation had a comparable risk of recurrences (pooled HR 1.30, 95% CI 0.91–1.85, *p* = 0.15).

**Figure 3:**
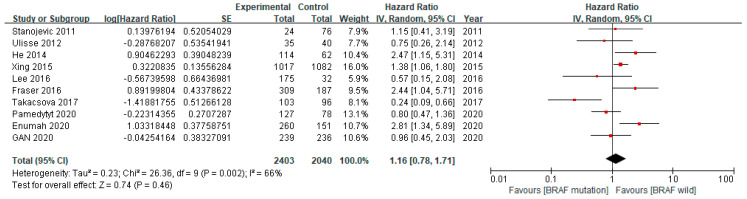
Forest plot of the included studies analyzing the adjusted recurrence rates between the patients with the BRAF V600E mutation and those with wild-type BRAF.

This comparable HR of the BRAF V600E mutation for recurrences remained non-significant (pooled HR 1.16, 95% CI 0.78–1.71) after adjustment for potential confounders, including age, sex, and tumor size (Figure 3).

### 2.3. Subgroup Analysis of the BRAFV600E Mutation in Tumor Recurrence and Clinic Features

Subgroup analysis was conducted in order to investigate potential sources of heterogeneity, which may affect the prognosis of PTC. We analyzed three factors that might affect the prevalence and the effects of the BRAF V600E mutation: geographical region (America, Asia, or Europe), American Joint Committee on Cancer stage (I or II), and patient age.

**Figure 4:**
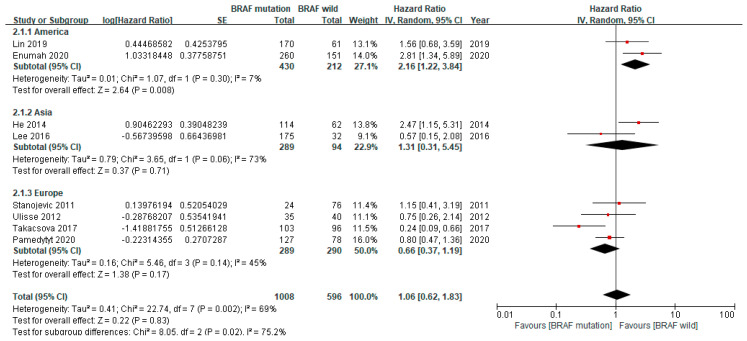
Forest plot of the subgroup analysis according to the geographical region.

Eight studies including 1604 patients reported the HR of the BRAF V600E mutation for recurrence according to the geographical region (Figure 4). The pooled HRs increased in both the America (HR 2.16, 95% CI 1.22–3.84) and Asia (HR 1.31, 95% CI 1.22–3.84) subgroups, although that for Europe showed no statistical significance (HR 0.66, 95% CI 0.37–1.19). Moderate heterogeneity (I^2^ = 73%, *p* = 0.06) was observed in the Asia subgroup, while the America (I^2^ = 7%, *p* = 0.30) and Europe (I^2^ = 45%, *p* = 0.14) subgroups showed low and insignificant heterogeneities, respectively.

**Figure 5:**
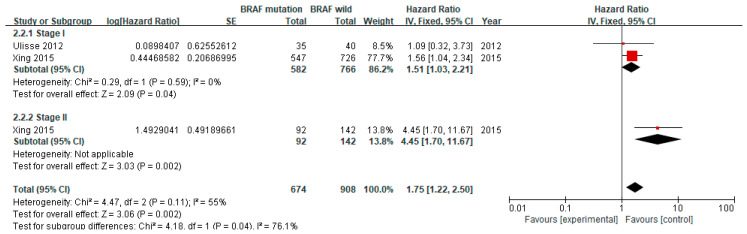
Forest plot of the subgroup analysis according to the tumor stage.

The recurrence rate of thyroid cancer in stage I was reported in two studies including 1273 patients, while that in stage II was found in one study of 234 patients (Figure 5). The BRAF V600E mutation significantly increased the risk of recurrence in both the stage I (pooled HR 1.51, 95% CI 1.03–2.21, *p* = 0.04) and the stage II PTC groups (HR 4.45, 95% CI 1.70–11.67, *p* = 0.01), respectively. No heterogeneity was found in the stage I subgroup (I^2^ = 0%, *p* = 0.59).

Three studies including 2773 patients reported HRs of recurrence by patient age. However, as all of these studies used different age categories, we cannot synthesize their results nor estimate an effect size.

### 2.4. Publication Bias

**Figure 6:**
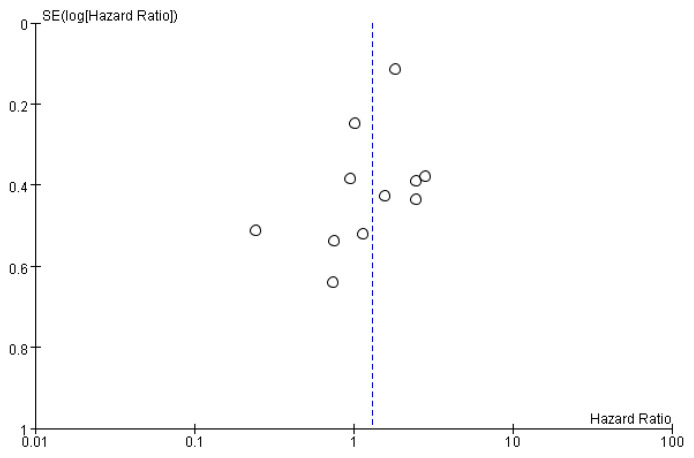
Funnel plot analysis demonstrating no publication bias.

A funnel plot analysis was performed to assess publication bias in the studies investigating recurrence rates (Figure 6). Both Egger’s (*p* = 0.173) and Begg’s (*p* = 0.139) statistics were non-significant. No publication bias was detected in the present meta-analysis.

## 3. Discussion

This updated and comprehensive meta-analysis demonstrates that a BRAF mutation does not increase the risk of recurrences in patients with PTC. BRAF mutations were first found in human cancers in 2002, and over 40 kinds of BRAF mutations have been identified since then [32]. The BRAF V600E mutation is the most frequent and specific genetic alteration found in PTC, which activates the tumorigenic mitogen-activated protein kinase signaling pathway [33,34]. The prevalence of the BRAF V600E mutation ranges from 22% to 85%, depending on the geographic location and iodine intake [35,36]. Most previous studies suggested that the BRAF V600E mutation is associated with an aggressive pathological type, extrathyroidal extension, advanced clinical stages, or the recurrence of PTC [14,15,16,17,18]. On the other hand, others have indicated that patients with the BRAF V600E mutation showed comparable recurrence rates or a similar clinical course [37,38,39,40]. We conducted a meta-analysis to address this controversy.

All previous meta-analyses except one found that the BRAF V600E mutation would increase the recurrence rate. Only Vuong et al. indicated that BRAF mutation was not associated with newly detected distant metastasis (pooled OR 1.26, 95% 0.80–1.98), although they used OR for outcome measurement [41]. Our results using HR confirmed that the BRAF V600E mutation is not associated with recurrence. Recent studies have indicated that the effect of the BRAF V600E mutation for PTC recurrence would be minimized, or even be protective, after adjustment of possible confounders including age, sex, tumor size, and multifocality [21,26,28]. Pamedytete et al. reported that the adjusted HR of the BRAF V600E mutation was 0.80 (95% CI 0.47–1.36), although the unadjusted HR was 1.01 (95% CI 0.62–1.64) for the recurrence of PTC [21]. In the present meta-analysis, the pooled HR of the enrolled studies also decreased from 1.30 (95% CI 0.91–1.85) to 1.16 (95% CI 0.78–1.71) after adjustment, which is consistent with the previous reports.

We performed subgroup analyses to identify potential sources of heterogeneity among the studies. Previous studies suggested that the effect of the BRAF V600E mutation was inconsistent according to the geographical region or ethnicity. Wang et al. reported that the HR for recurrences was variable among Americans (HR 2.1, 95% CI 1.54–2.86), Asians (HR 1.98, 95% CI 1.30–3.01), and Europeans (HR 1.83, 95% CI 1.36–2.45) [20]. In another meta-analysis, Caucasian patients with the BRAF V600E mutation had a 2.7 times higher risk of recurrences than those without the BRAF mutation, while Asians showed no significant increase regardless of their BRAF mutational status [17]. Our subgroup analysis of the geographical region of the included studies also found that patients in America had the highest risk of recurrences (HR 2.16, 95% CI 1.22–3.84), while those in Europe had the lowest risk (HR 0.66, 95% CI 0.37–1.19). All of these studies imply that the geographical region or ethnicity should be considered to interpret the effect of the BRAF V600E mutation on recurrence.

The BRAF V600E mutation is associated with an advanced stage of PTC, which may lead to a poor outcome. However, there are few studies about the association of the BRAF V600E mutation with recurrence in different stages. Ulisse et al. reported that the HR of the BRAF V600E mutation in stage I patients (HR 1.09, 95% CI 0.32–3.73) was higher than that in patients with all stages (HR 0.75, 95% CI 0.26–2.14) [30]. Conversely, Takacsova et al. found that the BRAF V600E mutation increased the HR for recurrence up to 24.5 as the N stage increased [25]. Xing et al. also indicated that the effect of the BRAF mutation for recurrence in 234 patients with stage II PTC (HR 4.45, 95% CI 1.70–11.67) was higher than that in 1371 patients with stage I PTC (HR 1.56, 95% CI 1.04–2.34) [28]. Our results for the subgroup analysis of stage were consistent with the findings of Xing et al. These results further suggest that the effect of the BRAF V600E mutation might be underestimated, as the number of patients with earlier stage PTC have increased.

The influence of the BRAF V600E mutation for recurrence among different age groups is controversial, although older age is a well-known risk factor of recurrence. A large, multicenter cohort study indicated an association between older age (≥45 years) and the BRAF V600E mutation to have poor clinical outcomes, including recurrences [28]. Shen et al. further found that the age of patients with the BRAF V600E mutation showed a positive linear association with increased mortality, while patients without BRAF mutations showed consistent mortality rates across age groups [42]. On the contrary, Gan et al. suggested that the BRAF V600E mutation was not predictive for recurrence in either <55 and ≥55 years age groups, respectively [23]. Takacsova et al. demonstrated that both younger (<35 years) and older (≥60 years) patients with the BRAF V600E mutation had a higher risk of recurrence, while middle-aged (35–60 years) patients with the BRAF mutation did not confer an additional risk compared with patients without the BRAF V600E mutation [25]. Differences in age categorization and geographical region may contribute to these conflicting results.

The present meta-analysis has some limitations. First, only 11 articles focusing on the HRs of recurrences were identified and included. Although we found around 50 studies reporting the recurrence rate according to the BRAF mutation status, we could not calculate the HR from those studies [19]. The exclusion of a relatively large number of studies could have resulted in some type of bias. Second, the effect of the BRAF V600E mutation can be altered by other mutations, including TERT mutations [43]. As recent studies have identified novel molecular markers, an updated meta-analysis would be needed in the near future. Third, we did not perform meta-regression or multivariate analysis, although there was moderate heterogeneity among studies. Because of the incompleteness of the data, we could only conduct subgroup analyses for possible confounders. Further studies are warranted to draw more precise conclusions.

## 4. Materials and Methods

### 4.1. Search Strategy

This meta-analysis was conducted in accordance with the recommendations of the preferred reporting items for systematic reviews and meta-analysis protocols (PRISMA-P) [44]. The following electronic databases were searched from inception to 31 May 2020: PubMed/MEDLINE (*n* = 899), SCOPUS (*n* = 920), the Cochrane Database of Systematic Reviews (*n* = 7), and the Web of Science Core Collection (*n* = 870). Two authors (H.K. and X.L.) independently performed the review using the search terms (“Thyroid Cancer” OR “PTC”), (“Recurrence” OR “Prognosis”), and (“BRAF”) using the Boolean “AND” operator.

### 4.2. Eligibility Criteria

The inclusion criteria were as follows: (1) studies that involved patients with PTC receiving thyroidectomy, (2) studies that included statistical data on BRAF mutations, (3) outcomes measured—hazard ratio of recurrences and/or persistent disease, and (4) no limitation for study design.

The exclusion criteria were as follows: (1) studies on patients with distant metastasis; (2) case reports, commentaries, and editorials; (3) nonhuman studies including experimental studies and animal studies; and (4) articles not written in English.

### 4.3. Data Extraction and Quality Assessment

Two reviewers carefully reviewed the full text of the eligible studies and independently extracted relevant information. Data including the names of authors, publication year, country, geographical region or ethnicity, study period, number of patients, BRAF mutation status, recurrence rates, and hazard ratio of recurrences were obtained with a structured data collection form. The Newcastle–Ottawa scale was used for the assessment of study quality [45]; studies with scores of 8 or higher were eligible for inclusion in this meta-analysis.

### 4.4. Statistical Analysis

Review Manager Version 5.3 (Cochrane Collaboration, Oxford, UK) was used to conduct all of the statistical calculations. Statistical heterogeneity among these studies was calculated by Cochran’s Q test and the I^2^ index (≤25% = insignificant heterogeneity, 26–50% = low heterogeneity, 51–75% = moderate heterogeneity, and over 75% = high heterogeneity). The random-effects model was used when moderate or greater heterogeneity was present among the studies; otherwise, the fixed-effects model was applied. Publication bias was assessed by Egger’s test and Begg’s test using funnel plots [46,47].

## 5. Conclusions

This updated meta-analysis confirmed that the BRAF V600E mutation does not increase the risk of recurrences in patients with PTC. Differences in geographical region or tumor stage should be considered to interpret the effect of BRAF mutations on the risk of recurrence.

## Figures and Tables

**Table 1:** Characteristics of the studies included in the meta-analysis.

First Author	Year	Country	Region	Study Period	No. of Cases	BRAF Mutation (%)	NOS
Pamedytyte [21]	2020	Lithuania	European	03–17	205	127 (62.0%)	9
Enumah [22]	2020	USA	American	00–07	411	260 (63.3%)	9
Gan [23]	2020	China	multiethnic	NR	475	239 (50.3%)	8
Lin [24]	2019	USA	American	73–09	231	170 (73.6%)	9
Takacsova [25]	2017	Slovakia	European	09–12	199	103 (51.8%)	8
Lee [26]	2016	Korea	Asian	07–14	207	175 (84.5%)	8
Fraser [27]	2016	Australia	Australian	90–12	496	309 (62.3%)	8
Xing [28]	2015	USA	multiethnic	78–11	2099	1017 (48.5%)	8
He [29]	2014	China	Asian	09–11	176	114 (64.8%)	8
Ulisse [30]	2012	Italy	European	NR	75	35 (46.7%)	8
Stanojevic [31]	2011	Serbia	European	92–06	100	24 (24.0%)	8

Abbreviations: NR, not recorded; NOS, Newcastle–Ottawa scale.
